# Study Protocol and Preliminary Results of the Impact of Occupational Health Workers' Activities on Their Health: Nationwide Prospective Internet-Based Survey

**DOI:** 10.2196/35290

**Published:** 2022-07-28

**Authors:** Kazunori Ikegami, Yasuro Yoshimoto, Hiroka Baba, Shingo Sekoguchi, Hajime Ando, Akira Ogami

**Affiliations:** 1 Department of Work Systems and Health Institute of Industrial Ecological Sciences University of Occupational and Environmental Health Fukuoka Japan

**Keywords:** Japan, occupational health, worker, internet surveys, questionnaires, cohort study, COVID-19, mental health, online health

## Abstract

**Background:**

Owing to the impact of the COVID-19 pandemic, work environments and systems, as well as occupational health measures or activities that fall within our research field, are constantly changing. It is necessary to assess the impact of these changes on the physical and mental health of workers.

**Objective:**

To assess how occupational health measures affect the health of workers, we conducted a baseline, longitudinal internet-based survey among Japanese workers in October 2021 and additionally scheduled 2 follow-up surveys for 2022 and 2023. We describe the details of the protocol of the work systems and health internet research (WSHIR) study, provide an overview of the results of the baseline survey, and discuss the study procedures and data used in the study.

**Methods:**

This prospective cohort study was conducted online among internet monitors. The baseline survey was conducted from October 1 to 7, 2021. This study targeted those who were working and between the ages of 20 and 69 years. A total of 5111 respondents who passed the screening survey and proceeded to the main survey were enrolled according to collection units organized by sex and age. For the screening and main surveys, the questionnaire consisted of 9 and 33 items with 9 and 55 questions, respectively. Consistency and completeness checks were performed after the questionnaires were submitted. We compared basic characteristics, such as sex, age group, educational background, and marital status, among all participants, including those who withdrew from the analysis.

**Results:**

Of the 5111 initial survey respondents, 571 (11.2%) were considered fraudulent. The data of the remaining 4540 (88.8%) participants (2273, 50.1%, males; 2267, 49.9%, females) included in the analysis were well balanced across participant sex and age groups according to the sampling plan because there was no significant difference by sex and age group using the chi-square test for checking the distribution bias of the participants (*P*=.84). Compared to female participants, male participants tended to be more likely to be managers and supervisors (323, 14.2%, males; 86, 3.8%, females), to work in a secondary industry (742, 32.6%, males; 357, 15.7%, females), and to have an annual income of ≥5 million yen (976, 42.9%, males; 429, 18.9%, females). For the evaluation of a psychological indicator, Kessler 6 (K6) score, by sex and age group, the characteristics of the score distribution of the included participants were similar to those reported in previous studies.

**Conclusions:**

This study presents a protocol and overview of the results of an internet-based occupational health survey of workers. Using the results of this survey, we hope to evaluate the changes in occupational health activities and their impact on workers' health while controlling for the COVID-19 pandemic.

## Introduction

The global outbreak of COVID-19 in 2020 had a profound impact on the economy, daily and working life, and medical practice in Japan [1-3]. The Japanese government repeatedly announced a state of emergency, asking the public to exercise voluntary restraint, such as refraining from going out and traveling to distant places, curtailing corporate business activities, and refraining from dinners and other socializing opportunities [4]. In the occupational field, several COVID-19 infection control guidelines were developed by various industries and organizations [5]. The introduction of telecommuting [6] and the implementation of COVID-19 vaccination or antigen testing in workplaces have also been recommended. These changes brought about by the COVID-19 pandemic have resulted in dramatic changes in the work environment, work systems, and occupational health activities [7,8].

In Japan, the occupational health system is defined using occupational safety and health laws, and occupational health services are implemented in many companies [9,10]. Companies with 50 or more employees are required to appoint at least 1 industrial physician and 1 health manager [9]. Occupational physicians have been among the leaders in promoting infection and prevention measures in workplaces during the COVID-19 pandemic [5]. Occupational physicians have often played a central role in workplace COVID-19 vaccination programs as well as in awareness-raising activities for countermeasures against the COVID-19 pandemic in occupational fields. They provide health support for employees affected by COVID-19 and health management for all employees in view of the ever-changing landscape and the impact of the COVID-19 pandemic. We believe that the COVID-19 pandemic brought a more vivid focus on occupational physicians by the public and that it served as the turning point for the promotion of occupational health activities.

In Japan, since the lifting of the government's emergency restrictions at the end of October 1, 2021, until now (end of June 2022), no restrictions have been in place, and more than 60% of the population has completed the third vaccination against COVID-19. It is possible that COVID-19 will once again become prevalent in Japan and that countermeasures will have to be taken on a case-by-case basis. However, it is unlikely that the situation will change as dramatically as it did between early 2020 and September 2021. Looking ahead to the post–COVID-19 pandemic era, it is important to monitor future occupational health activities and assess how they will affect the work environment and systems or the health of workers.

We consider it necessary to focus on future challenges and issues regarding occupational health fields by looking ahead at the post–COVID-19 pandemic period. Such future challenges and issues include cooperation between health management and practice; workers’ health management, including annual health checkups, countermeasures against communicable diseases in the workplace, and fitness to work; and the actual status of occupational health services and occupational physician activities, in addition to longitudinally assessing how these affect the health of workers. Therefore, we conducted a longitudinal study, that is, a work systems and health internet research (WSHIR) study, among workers from October 2021. In addition, we scheduled 2 follow-up surveys for 2022 and 2023.s

The aim of this paper is to present details of the WSHIR study protocol. Moreover, it provides an overview of the results of the baseline survey and includes a discussion of the study procedures and improvements to the quality of the data used in the study. We plan to use the data from this study to inform various research themes focused on occupational health issues, such as the impact of occupational health services and activities on workers, changes, and new challenges for occupational health in the workplace in the post–COVID-19 state.

## Methods

### Study Design

This survey was a prospective cohort study conducted online among internet monitors registered with Cross Marketing Inc. (Tokyo, Japan), which is a Japanese internet research contractor with 4.7 million registered monitors. We sent participation information to the registered monitors, so this was not an open survey.

The baseline survey was conducted from October 1 to 7, 2021. Two follow-up surveys are scheduled for 2022 and 2023. The study targeted those who were working and between the ages of 20 and 69 years in the baseline survey.

A document describing the time required to complete the survey, storage location, period of the survey data, the investigator, and the purpose of the study are available on the website of the Department of Work System and Health, Institute of Industrial Ecological Sciences, University of Occupational and Environmental Health, Japan (in Japanese).

### Ethical Considerations

All participants provided informed consent online to participate in this study. The study was approved by the ethics committee of the University of Occupational and Environmental Health, Japan (reference no. R3-037).

### Sample Size

The statistical method was not determined, because this study was mainly exploratory. However, the sample size was calculated by adapting the following conditions, which are most likely to be assumed: in sectional or cohort studies, 2-sided significance level (1 – α)=95; power (1 – β, percentage chance of detecting)=80; ratio of sample size, unexposed/exposed=1; percentage of unexposed with the outcome=5; odds ratio=1.5; risk/prevalence ratio=1.5; and risk/prevalence difference=2.3. Under these conditions, the required total sample size was calculated to be 3380 [11]. Hence, we set the sample size to 5000 to account for data exclusion.

### Sampling Plan

Only people registered with Cross Marketing Inc. (Tokyo, Japan) could complete the survey. First, the questionnaire for the screening survey confirmed informed consent to participate in the study; the respondents were regular workers, we excluded temporary and part-time workers, and the age was ≥20 years. Only respondents who met these conditions proceeded to the main survey, which consisted of 10 collection units organized by both sex and age group, with 500 respondents per collection unit, for a total sample size of 5000. Third, each collection unit was designed to be closed once it reached 520 respondents; thereafter, respondents could not proceed from the screening stage to the main survey stage.

Personal information was not collected from a series of surveys. The respondents were contracted according to the privacy policy of Cross Marketing, Inc.

### Recruitment Process for Participants

As instructed, Cross Marketing Inc. sent emails that included a link to the website, along with an introduction to the survey and an entry to the questionnaire page. There is an automatic method for capturing the responses on a website. Completion of this survey was voluntary, and as an incentive to complete the survey, the respondents earned points that could be exchanged for various products.

We counted the number of participants in this survey by counting the monitor IDs assigned to the respondents when they accessed the survey system. For the first survey, participation invitations were sent via email to approximately 59,000 monitors randomly selected by Cross Marketing Inc. from among more than 5 million registered monitors. This survey was started on October 1, 2021, and all sample collections were completed on October 5, 2021.

### Measurements

For the screening and main surveys, the questionnaire consisted of 9 and 33 items with 9 and 55 questions, respectively. The questionnaire consisted of 33 pages, with 1 item per page. The survey consisted of the following 3 major categories: basic and socioeconomic characteristics and health status, a psychological questionnaire that was already validated, and questions pertaining to occupational medicine and health.

Questions relating to basic and socioeconomic characteristics included sex, age, marital status, income, educational background, area of residence, and work-related factors, such as occupation, number of employees at workplaces (branches, factories, and sales offices), type of industry where the participants worked, and average working hours. In addition, single items regarding the participants’ health status included present medical history, presence of current physical and psychological problems and their causes, and the number of days of sick leave absence.

The psychological questionnaires included the Brief Job Stress Questionnaire [12], the Japanese version of the 3-item Utrecht Work Engagement Scale [13,14], and Quantity and Quality [15] as an evaluation index of presenteeism. Other psychological questionnaires were the Patient Health Questionnaire-2 [16] and the Kessler 6 (K6) score [17,18]. The reliability and validity of these questionnaires on psychological scales have been confirmed in previous studies. The Japanese versions of these psychological scales were used without modification.

For questions related to occupational medicine and health, we surveyed the perceived workplace health support [19], the actual state of health management for workers (eg, health checkups, countermeasures for return to work), fitness to work, countermeasures against communicable diseases in the workplace, provision of occupational health services, health consultation services, and management of workers’ health information. Each question consisted of 1 or 2 original items and was not evaluated by calculating the scale scores.

All the aforementioned questionnaires were created or selected by 3 experts certified by senior occupational health physicians, who were certified by the Japan Society for Occupational Health, the body that discusses current issues regarding occupational health in Japan. After generating the questionnaires, we requested 3 other occupational physicians to respond and review the drafts.

Additionally, we verified that these questions could be answered without any problems on a web-based system before conducting the main survey. We also checked for inappropriate expressions, ease of answering, typographical errors, and other issues that were used to revise the questionnaires.

### Completeness Check

Consistency and completeness checks were performed after the questionnaires were submitted. We detected fraudulent respondents based on the 3 types of algorithms designed in this survey:

- Respondents who failed to correctly answer 2 basic knowledge questions unrelated to the survey. Specifically, one was to correctly select odd numbers among five 2-digit numbers, and the other was to correctly select multiples of 3 out of five 2-digit numbers.
- Respondents who provided contradictory answers to the 3 predetermined questions (the 3 questions were likely to be contradictory if the respondents did not answer them carefully). Specifically, we designed a 2-option (yes/no) question on whether the respondent had undergone a health checkup within the past year, followed by a 6-option question on how to obtain, store, and use the results of the health checkup. In 1 instance, the respondent answered, “I had not had a health checkup within a year,” but the respondent also answered, “I kept the results of the health checkup within a year.”
- Respondents whose response time was <3 minutes. To exclude questionnaires submitted too soon, we excluded those with a response time of less than 3 minutes. We set these cut-off points based on our actual response time to the questionnaires and the fact that Fujino et al [20] set the cut-off points at 6 minutes for the questionnaire consisting of 55 items and 160 questions in their study.

Respondents who met the exclusion criteria based on these consistency or completeness checks were considered withdrawn; otherwise, they were considered enrolled.

### Statistical Analysis

We compared basic characteristics, such as sex, age group, educational background, and marital status, between enrolled and withdrawn participants in the analysis using the chi-square test. In addition, we used the chi-square test to compare the characteristics between respondents who provided contradictory answers to the 3 questions and those who did not and between those whose response time was <3 minutes and those whose response time was ≥3 minutes. The comparisons between those who answered the 2 basic knowledge questions correctly and incorrectly were not analyzed because of incorrect answers.

We analyzed the educational background, marital status, occupation, industrial classification, number of employees in the business unit where the participants worked, number of employees in the company where the participants worked, and annual income (yen) by sex or age group (20-29, 30-39, 40-49, 50-59, and 60-69 years) using the chi-square test. We analyzed the K6 score by sex using the Mann-Whitney *U* test or by age group using the Kruskal-Wallis test.

## Results

### Participant Details

**Figure 1:**
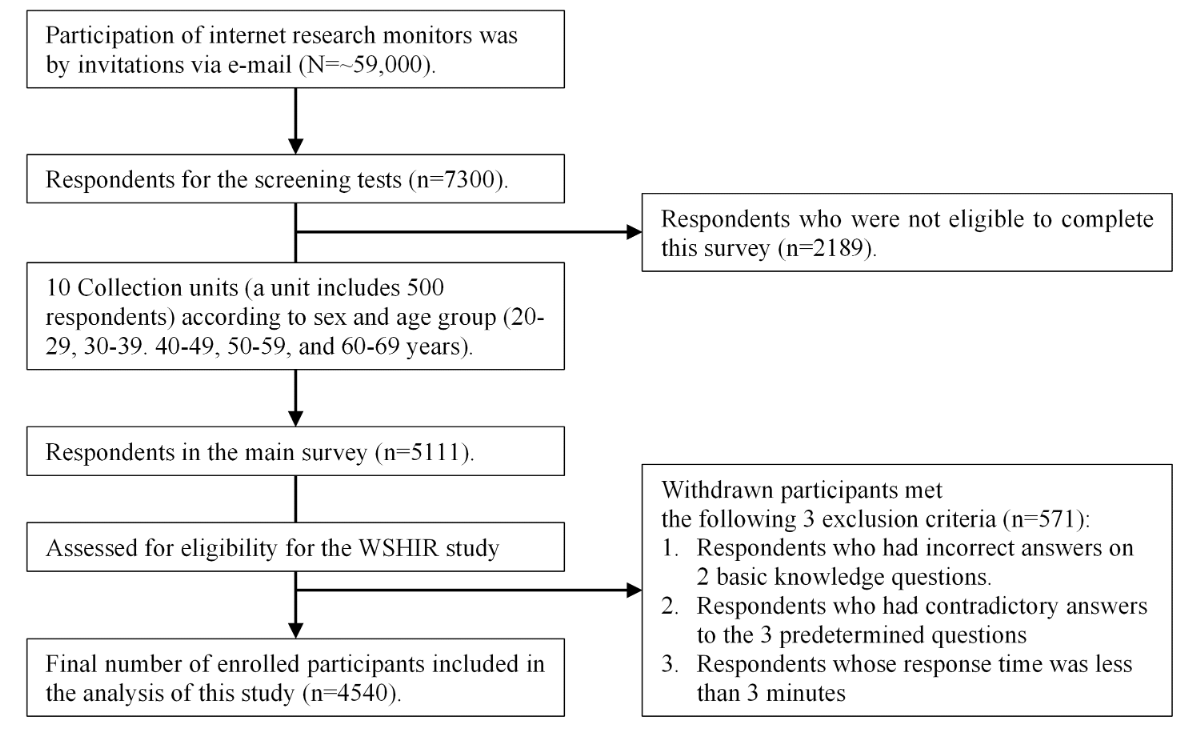
Flowchart of participant selection. WSHIR: work systems and health internet research.

As shown in Figure 1, the survey invitation was sent to approximately 59,000 registrants, and 7300 (12.4%) responded to the screening survey. A total of 2189 (30%) respondents were excluded from the screening survey stage, and 5111 (70%) completed the main survey (completion rate=ratio of users who completed the survey to users who agreed to participate). The distribution of the enrolled participants by sex and age group (ie, by 10 collection units) is shown in Table 1. The number of enrolled participants by sex and age group was evaluated using the chi-square test to check for distribution bias; there was no significant difference (*P*=.99). Of the 5111 respondents, 571 (11.2%) withdrew. Of those with duplication, 434 (8.5%) provided contradictory answers to the 3 questions, 161 (3.2%) had a response time of less than 3 minutes, and none had incorrect answers to the 2 basic knowledge questions. The final number of participants enrolled in the analysis was 4540 (Figure 1). The number of enrolled participants by sex and age group was evaluated using the chi-square test to check for distribution bias; there were no significant differences (*P*=.84).

According to residence (prefecture), the 4540 participants were distributed across all 47 prefectures in Japan. The highest and lowest proportion of respondents per 100,000 population was 5.4 (Tokyo) and 1.5 (Miyazaki Prefecture), with a 47-prefecture median (quartile) of 3.0 (2.5-3.6). Among 9 (19.1%) of the 47 prefectures, with a population of more than 5 million per prefecture, 7 (77.8%) were among the top 10 with the highest proportion of respondents per 100,000 population.

We compared the basic characteristics of the enrolled and withdrawn participants (Table 2). There were no significant differences in sex, educational background, or marital status among groups. However, a higher proportion of younger participants withdrew.

We also compared the basic characteristics of the respondents who provided contradictory answers to the 3 questions (Table 2). The number of respondents who provided contradictory answers was 434 (8.5%) of 5111. They tended to be younger and less educated, and 24 (5.5%) had a response time of <3 minutes; in addition, they were significantly more in number (n=434, 8.5%) than those who provided no contradictory responses (n=137, 2.9%).

Next, we compared the basic characteristics of respondents whose response times were ≥3 and <3 minutes (Table 3). The number of respondents with a response time of <3 minutes was 161 (3.2%). The median, 25th, and 75th percentiles were 6 minutes 49 seconds, 4 minutes 56 seconds , and 9 minutes 50 seconds, respectively. Those with a response time of <3 minutes tended to be younger, better educated, and unmarried. Of those with a response time of <3 minutes, those with contradictory answers were significantly more in number (n=24, 14.9%) than those with a response time of ≥3 minutes (n=410, 8.3%).

We compared the basic and work-related characteristics of the enrolled participants between the sexes and among the 5 age groups (Tables 4 and 5). Male participants tended to be more likely to be married and have a university or graduate school degree than female participants. In terms of work-related characteristics, male participants tended to be more likely to be managers and supervisors, work in a secondary industry or in large-size workplaces or enterprises, and have an annual income of ≥5 million yen (US $36,183.51) than the female participants. Female participants were significantly more likely to be in a third industry, to work in small workplaces or enterprises, and to have an annual income of <2.99 million yen (US $20,986.44) than the male participants.

The 20-29-year age group tended to be less likely to be married compared to those in other age groups. Regarding educational background, younger participants tended to be more likely to have a university or graduate school degree, be regular employees, and be employed in large workplaces or enterprises. With the increasing age of the respondents, an increased proportion was found among those who were engaged in workplaces or enterprises (49 employees or fewer), as well as those who were engaged in the secondary industry.

The K6 score was higher in female participants than in male participants; in addition, the K6 score tended to be higher in those aged 20-39 years (Table 6).

**Table 1:** Respondents and enrolled participants per collection unit by age group and sex.

Age group (years)	All participants (N=5111), *P*=.99		Enrolled participants (N=4540), *P*=.84	
	Male participants (N=2551), n (%)	Female participants (N=2560), n (%)	Male participants (N=2273), n (%)	Female participants (N=2267), n (%)
20-29	507 (19.9)	513 (20.0)	425 (18.7)	418 (18.4)
30-39	505 (19.8)	509 (19.9)	420 (18.5)	448 (19.8)
40-49	514 (20.1)	500 (19.5)	467 (20.5)	448 (19.8)
50-59	511 (20.0)	518 (20.2)	480 (21.1)	481 (21.2)
60-69	514 (20.1)	520 (20.3)	481 (21.2)	472 (20.8)

**Table 2:** Comparison of enrolled and withdrawn participants.

Characteristics			Total (N=5111), n (%)		Participants			Contradictory answers^a^	
				Enrolled (N=4540), n (%)		Withdrawn (N=571), n (%)	No (N=4677), n (%)		Yes (N=434), n (%)
**Sex; participants *P*=.53, contradictory answers *P*=.33**									
	Male	2551 (49.9)		2273 (50.1)		278 (48.7)	2344 (50.1)		207 (47.7)
	Female	2560 (50.1)		2267 (49.9)		293 (51.3)	2333 (49.9)		227 (52.3)
**Age group (years); participants *P*<.001, contradictory answers *P*<.001**									
	20-29	1020 (20.0)		843 (18.6)		177 (31.0)	898 (19.2)		122 (28.1)
	30-39	1014 (19.8)		868 (19.1)		146 (25.6)	914 (19.5)		100 (23.0)
	40-49	1014 (19.8)		915 (20.2)		99 (17.3)	941 (20.1)		73 (16.8)
	50-59	1029 (20.1)		961 (21.2)		68 (11.9)	967 (20.7)		62 (14.3)
	60-69	1034 (20.2)		953 (21.0)		81 (14.2)	957 (20.5)		77 (17.7)
**Educational background; participants *P*=.83, contradictory answers *P*=.04**									
	Junior high school or high school	1104 (21.6)		976 (21.5)		128 (22.4)	995 (21.3)		109 (25.1)
	Technical college or junior college	1086 (21.2)		963 (21.2)		123 (21.5)	984 (21.0)		102 (23.5)
	University or graduate school	2921 (57.2)		2601 (57.3)		320 (56.0)	2698 (57.7)		223 (51.4)
**Marital status; participants *P*=.11, contradictory answers *P*=.62**									
	Unmarried	2486 (48.6)		2190 (48.2)		296 (51.8)	2270 (48.5)		216 (49.8)
	Married	2625 (51.4)		2350 (51.8)		275 (48.2)	2407 (51.5)		218 (50.2)
**Contradictory answers^a^**									
	No	4677 (91.5)		4540 (100.0)		137 (24.0)	N/A^b^		N/A
	Yes	434 (8.5)		0		434 (76.0)	N/A		N/A
**Response time (minutes; contradictory answers *P*=.003)**									
	≥3	4950 (96.8)		4540 (100.0)		410 (71.8)	4540 (97.1)		410 (94.5)
	<3	161 (3.2)		0		161 (28.2)	137 (2.9)		24 (5.5)

^a^Respondents who provided contradictory answers to the 3 predetermined questions (yes) and those who did not (no).

^b^N/A: not applicable.

**Table 3:** Basic characteristics of respondents (N=5111) whose response times were ≥3 and <3 minutes.

Characteristics		Total, n (%)		Response time (minutes)		
				≥3 (N=4950), n (%)		<3 (N=161), n (%)
**Sex; *P*=.56**						
	Male	2551 (49.9)	2467 (49.8)		84 (52.2)	
	Female	2560 (50.1)	2483 (50.2)		77 (47.8)	
**Age group (years); *P*<.001**						
	20-29	1020 (20.0)	957 (19.3)		63 (39.1)	
	30-39	1014 (19.8)	960 (19.4)		54 (33.5)	
	40-49	1014 (19.8)	983 (19.9)		31 (19.3)	
	50-59	1029 (20.1)	1021 (20.6)		8 (5.0)	
	60-69	1034 (20.2)	1029 (20.8)		5 (3.1)	
**Educational background; *P*=.004**						
	Junior high school or high school	1104 (21.6)	1082 (21.9)		22 (13.7)	
	Technical college or junior college	1086 (21.2)	1059 (21.4)		27 (16.8)	
	University or graduate school	2921 (57.2)	2809 (56.7)		112 (69.6)	
**Marital status; *P*=.02**						
	Unmarried	2486 (48.6)	2393 (48.3)		93 (57.8)	
	Married	2625 (51.4)	2557 (51.7)		68 (42.2)	
**Contradictory answers^a^; *P*>=.003**						
	No	4677 (91.5)	4540 (91.7)		137 (85.1)	
	Yes	434 (8.5)	410 (8.3)		24 (14.9)	

^a^Respondents who provided contradictory answers to the 3 predetermined questions (yes) and those who did not (no).

**Table 4:** Basic characteristics of enrolled participants by sex.

Characteristics		Male participants (N=2273), n (%)	Female participants (N=2267), n (%)
**Educational background; *P*<.001**			
	Junior high school or high school	501 (22.0)	475 (21.0)
	Technical college or junior college	276 (12.1)	687 (30.3)
	University or graduate school	1496 (65.8)	1105 (48.7)
**Marital status; *P*<.001**			
	Unmarried	877 (38.6)	1313 (57.9)
	Married	1396 (61.4)	954 (42.1)
**Occupation; *P*<.001**			
	Regular employees	1257 (55.3)	1354 (59.7)
	Managers	323 (14.2)	86 (3.8)
	Others	693 (30.5)	827 (36.5)
**Industrial classification; *P*<.001**			
	Primary industry	8 (0.4)	3 (0.1)
	Secondary industry	742 (32.6)	357 (15.7)
	Third industry	1523 (67.0)	1907 (84.1)
**Number of employees of business units where the participants worked; *P*<.001**			
	1-49	809 (35.6)	1028 (45.3)
	50-999	831 (36.6)	726 (32.0)
	≥1000	561 (24.7)	375 (16.5)
	Unclear	72 (3.2)	138 (6.1)
**Number of employees of companies where the participants worked; *P*<.001**			
	1-49	643 (28.3)	818 (36.1)
	50-299	457 (20.1)	445(19.6)
	300-999	292 (12.8)	269 (11.9)
	1000-9999	466 (20.5)	290 (12.8)
	≥10,000	274 (12.1)	191 (8.4)
	Unclear	141 (6.2)	254 (11.2)
**Annual income (yen); *P*<.001**			
	<3 million (<US $21,710.11^a^)	281 (12.4)	713 (31.5)
	3-4.9 million (US $21,710.11-$35,459.84)	735 (32.3)	720 (31.8)
	5-9.9 million (US $36,183.51-$71,643.35)	787(34.6)	354 (15.6)
	≥10 million (US $72,367.02)	189 (8.3)	75 (3.3)
	Unclear	281 (12.4)	405 (17.9)

^a^An exchange rate of 1 Japanese yen=US $0.0072 has been applied.

**Table 5:** Basic characteristics of enrolled participants by age group.

Characteristics			20-29 years (N=843), n (%)		30-39 years (N=868), n (%)		40-49 years (N=915), n (%)		50-59 years (N=961), n (%)		60-69 years (N=953), n (%)
**Educational background; *P*<.001**											
	Junior high school or high school	145 (17.2)		147 (16.9)		197 (21.5)		281 (29.2)		206(21.6)	
	Technical college or junior college	144 (17.1)		150 (17.3)		221 (24.2)		251 (26.1)		197 (20.7)	
	University or graduate school	554 (65.7)		571 (65.8)		497 (54.3)		429 (44.6)		550 (57.7)	
**Marital status; *P*<.001**											
	Unmarried	632 (75.0)		436 (50.2)		443 (48.4)		389 (40.5)		290 (30.4)	
	Married	211 (25.0)		432 (49.8)		472 (51.6)		572 (59.5)		663 (69.6)	
**Occupation; *P*<.001**											
	Regular employees	635 (75.3)		621 (71.5)		563 (61.5)		451 (46.9)		341 (35.8)	
	Managers	9 (1.1)		38 (4.4)		111 (12.1)		159 (16.5)		92 (9.7)	
	Others	199 (23.6)		209 (24.1)		241 (26.3)		351 (36.5)		520 (54.6)	
**Industrial classification; *P*=.01^a^**											
	Primary industry	4 (0.5)		3 (0.3)		2 (0.2)		2 (0.2)		0	
	Secondary industry	202 (24.0)		224 (25.8)		232 (25.4)		256 (26.6)		185 (19.4)	
	Third industry	637 (75.6)		641 (73.8)		681 (74.4)		703 (73.2)		768 (80.6)	
**Number of employees in business units where the participants worked; *P*<.001**											
	1-49	223 (26.5)		286 (32.9)		331 (36.2)		445 (46.3)		552 (57.9)	
	50-999	357 (42.3)		347 (40.0)		346 (37.8)		283 (29.4)		224 (23.5)	
	≥1000	197 (23.4)		186 (21.4)		199 (21.7)		202 (21.0)		152 (15.9)	
	Unclear	66 (7.8)		49 (5.6)		39 (4.3)		31 (3.2)		25 (2.6)	
**Number of employees in companies where the participants worked; *P*<.001**											
	1-49	132 (15.7)		209 (24.1)		268 (29.3)		375 (39.0)		477 (50.1)	
	50-299	211 (25.0)		199 (22.9)		181 (19.8)		179 (18.6)		132 (13.9)	
	300-999	135 (16.0)		127 (14.6)		126 (13.8)		91 (9.5)		82 (8.6)	
	1000-9999	166 (19.7)		136 (15.7)		174 (19.0)		156 (16.2)		124 (13.0)	
	≥10,000	93 (11.0)		111 (12.8)		86 (9.4)		95 (9.9)		80 (8.4)	
	Unclear	106 (12.6)		86 (9.9)		80 (8.7)		65 (6.8)		58 (6.1)	
**Annual income (yen); *P*<.001**											
	<3 million (<US $21,710.11^b^)	219 (26.0)		178 (20.5)		160 (17.5)		205 (21.3)		232 (24.3)	
	3-4.9 million (US $21,710.11-$35,459.84)	406 (48.2)		312 (35.9)		258 (28.2)		218 (22.7)		261 (27.4)	
	5-9.9 million (US $36,183.51-$71,643.35)	97 (11.5)		234 (27.0)		310 (33.9)		279 (29.0)		221 (23.2)	
	≥10 million (US $72,367.02)	11 (1.3)		36 (4.1)		54 (5.9)		82 (8.5)		81 (8.5)	
	Unclear	110 (13.0)		108 (12.4)		133 (14.5)		177 (18.4)		158 (16.6)	

^a^In 33% of the cells, the expected frequencies are <5; therefore, this *P* value is not accurate.

^b^An exchange rate of 1 Japanese yen=US $0.0072 has been applied.

**Table 6:** K6^a^ scores of enrolled participants by sex and age group (*P*<.001).

Characteristics		K6 score	Participants, n (%)
**Sex**			
	Male (N=2273)	0-7	2 (0.1)
	Female (N=2267)	0-7	3 (0.1)
**Age group (years)**			
	20-29 (N=843)	0-8	8 (0.9)
	30-39 (N=868)	0-9	9 (1.0)
	40-49 (N=915)	0-7	7 (0.7)
	50-59 (N=961)	0-6	6 (0.6)
	60-69 (N=953)	0-4	4 (0.4)

^a^K6: Kessler 6.

## Discussion

### Principal Findings

In October 2021, after the fifth wave of the COVID-19 pandemic had subsided in Japan, we conducted an internet-based occupational health survey among workers. Internet surveys have become more common in recent years in the fields of public health and epidemiology, health care services, and even medicine because of the potential to collect relatively large amounts of data in a short period [21]. Compared to conventional population and workplace surveys, internet surveys have the advantages of making it easier to achieve the target sample size, incorporating many conditions, surveying in a short period, and making it easier to conduct surveys targeting workers. We believe that our data obtained using the WSHIR study will be valuable for future research on the working conditions and health status of workers post–COVID-19.

One of the problems with internet surveys is that respondents may provide fraudulent answers [22,23]. Many private internet survey companies set up incentives, such as points that can be exchanged for products, to increase the number of registrants and encourage them to completely respond to various surveys. There is a possibility that some respondents may answer the questionnaires inappropriately without understanding the aims of the survey or the questionnaire just to obtain these incentives. Therefore, internet surveys must be designed to detect fraudulent respondents.

In this study, we used 3 algorithms to detect fraudulent respondents. The first was the setting of 2 basic knowledge questions that were not related to the main survey. However, all the respondents answered these questions correctly. It is possible that in many surveys, questions have already been prepared to detect fraudulent respondents or that many respondents are aware that the questions are designed to detect fraudulent practices; thus, respondents decide to respond correctly. However, we speculate that ensuring that the respondents are aware that the questionnaire contains algorithms to detect fraudulent respondents may have a deterrent effect on fraudulent responses.

Second, the way the 3 predetermined questions were set could lead to contradictory answers if they were not answered carefully. This algorithm is complicated because the respondent must be consistent among the 3 predetermined questions. In fact, 434 of 5111 respondents provided contradictory answers, and all were treated as fraudulent respondents.

Finally, the cut-off points were set to exclude premature response times to the questionnaire, which were recorded using a questionnaire system by Cross Marketing Inc. In this survey, the median, 25th, 75th, and 5th percentiles were 6 minutes 49 seconds, 4 minutes 56 seconds, 9 minutes 50 seconds, and 3 minutes 16 seconds, respectively. Therefore, we believe it was reasonable to exclude 161 respondents whose answers were within 3 minutes. Regarding the 2 conditions for detecting fraudulent respondents, we found that those who met one of the conditions were significantly more likely to meet the other condition.

This study found that respondents with extremely short response times tend to provide contradictory responses. In addition, this tendency was observed in younger participants. We speculate that 1 of the reasons for this is the possibility that there are a certain number of internet monitors who are only interested in obtaining incentives. When conducting a questionnaire survey with slightly more difficult content via the internet, as in this study regarding occupational health fields, the researchers propose that a procedure is needed to validate the data set provided by internet research contractors.

In this survey, the respondents’ residences were not added to the collection unit. However, we could enroll participants from all 47 prefectures in Japan, although the proportion of respondents tended to be higher in metropolitan areas. In a conventional occupational health survey, where the researcher directly asks for research cooperation from companies, it is almost impossible to adjust for this kind of regional distribution of participants, and we believe that the data are well balanced.

By examining the relationships among the variables, we confirmed the authenticity of the data. For example, compared to female participants, male participants had a higher proportion of managers and those engaged in the secondary industry as well as a higher annual income. In addition, regarding psychological indicators, it has been reported that the K6 scores of women are higher than those of men [24,25], and a similar trend was observed in this study. Regarding age groups, the younger age group was more likely to be unmarried, and the proportion of managers was higher in the 40-49- and 50-59-year age groups and lower in the 60-69-year age group. In Japan, most companies have a mandatory retirement age of 60 years, and those aged ≥60 years are often rehired without job positions, on contract, or in fixed-term employment. As for psychological indicators, previous studies have reported that K6 scores tend to be higher in younger persons, and similar results were observed in this study [26].

Selection bias is unavoidable in internet surveys. For example, those who use the internet and are willing to answer the questionnaire will inevitably be selected. Respondents were simply those who were registered with an internet research company and did not represent a particular population. *Measurement error* refers to errors caused by differences in the approach used in responding, even by the same respondent, depending on whether the answers are recorded using other methods (eg, by telephone or interview) or are self-recorded (eg, by the respondent self-completing individually) and whether the answers are shown once the questionnaire paper has been filled out or shown on an internet screen [22,27,28]. To improve the validity of this study, we adhered to the Checklist for Reporting Results of Internet E-Surveys (CHERRIES) statement [21]. In addition, it is important to clarify the characteristics of the survey population by comparing various factors with those in previous studies. This study focused on occupational health, and we confirmed work-related factors, such as occupation, industry classification, size of the workplaces or enterprises where the participants worked, annual income, working hours, occupational health system, and activity status in as much detail as possible. We also examined several health-related factors of workers and psychosocial indicators related to work that have been used in many previous studies, which can be compared to the results of this study.

This study is intended to be conducted over a 3-year period, starting in October 2021, when several people in Japan have been vaccinated against COVID-19, with the number of infected people remaining low even after the government lifted the state of emergency. Of course, there is a possibility of a recurrent epidemic in the future. However, the government and the Japan Federation of Economic Organizations (Nippon Keidanren) are making efforts to resume or strengthen economic activities, since the situation may be approaching a possible control of the COVID-19 pandemic [21,22]. Once controlled, this study could provide an overview of changes in occupational health in Japan and how COVID-19 has affected workers.

### Limitations

We mentioned some research limitations before, but there are several other limitations to this study. First, the sampling plan was not specifically designed to consider the unit of workplace characteristics, such as workplace size, location, and type of industry. Therefore, it should be noted that the results of this study generalize to the whole working population in Japan. Second, some of the participants belonged to the same workplace. According to the Statistics Bureau of the Ministry of Internal Affairs and Communications, the number of workplaces in Japan is approximately 578,000. The likelihood that all participants had different workplaces was approximately 16.8% by a simple calculation. Because of the possibility that participants may belong to the same workplace, we need to be careful when analyzing and evaluating the study data. However, as we plan to continue this study in the future, we believe that the quality of the data can be improved by obtaining data such as the zip codes of the workplaces in a follow-up survey. Third, all data in this survey were based on parameters self-reported by individual workers. Respondents of this study might be unaware of or might not correctly understand the health-related measures implemented in their workplaces, depending on their position or status. In addition, this study did not use objective indicators of mental or physical health. Such research should utilize many indicators that have been commonly used in previous studies and should be examined with reference to previous studies.

### Conclusion

We commenced an internet-based occupational health survey focusing on occupational health activities and workers' health in October 2021 when approximately 80% of the population aged ≥12 years had been vaccinated and the fifth wave of the COVID-19 pandemic was under control in Japan. This paper presents the protocol of this study and provides an overview of the data from the baseline survey, the study procedures, and the quality of the data in this survey. Using the data of this survey, we aimed to evaluate the changes in occupational health activities and their impact on workers' health after the COVID-19 pandemic was controlled. We plan to analyze the data from multiple perspectives and present new findings regarding occupational health fields sequentially.

## Abbreviations

K6: Kessler 6
WSHIR: work systems and health internet research
